# Supplementary figures and images for: Application of attenuated total reflection–Fourier transform infrared spectroscopy in semi-quantification of blood lipids and characterization of the metabolic syndrome
Source: PLoS One. 2025 Jan 30;20(1):e0316522. doi: 10.1371/journal.pone.0316522 (PMC11781649; doi:10.1371/journal.pone.0316522)

## Appendix 1. Distribution of blood lipid values in MetS and non-MetS groups

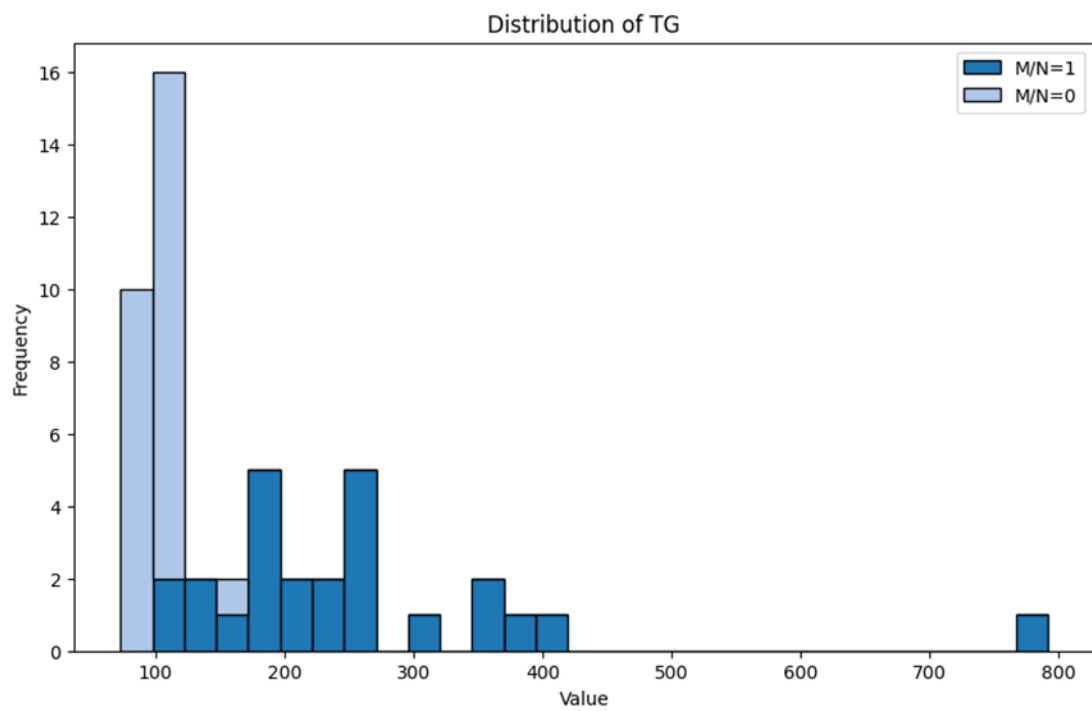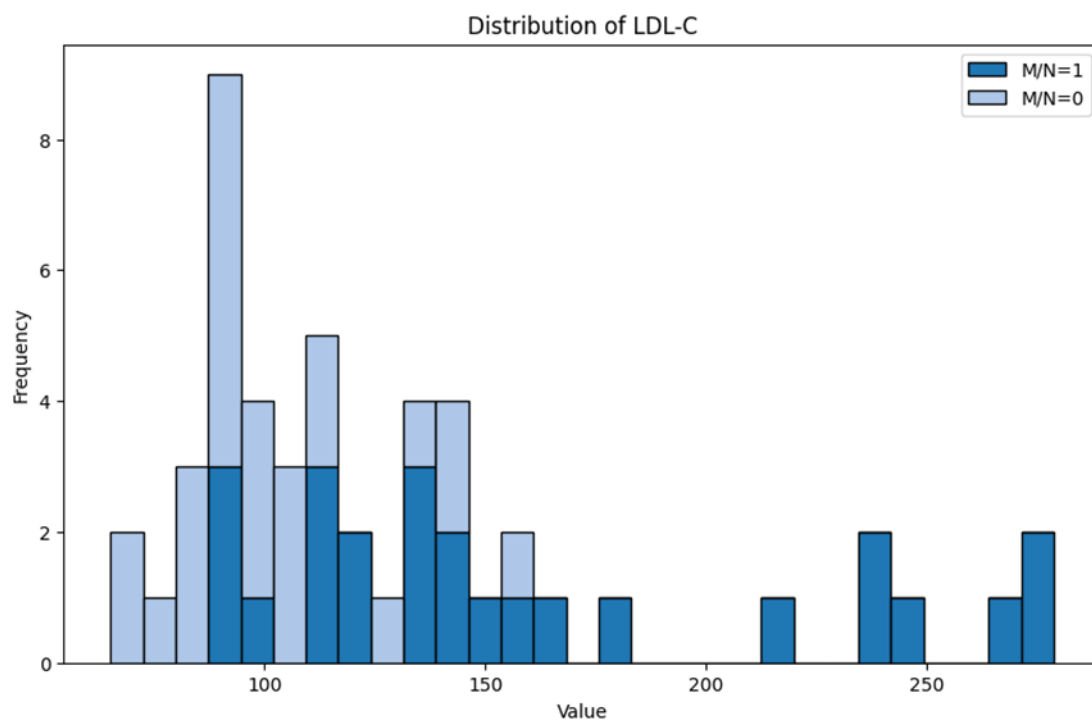

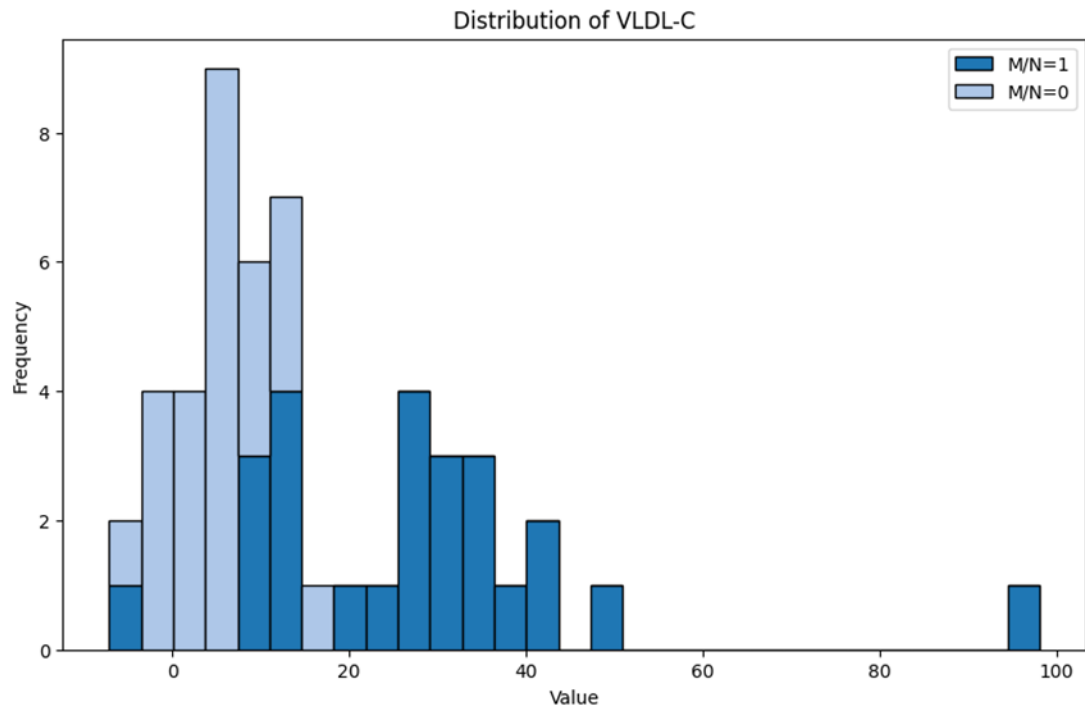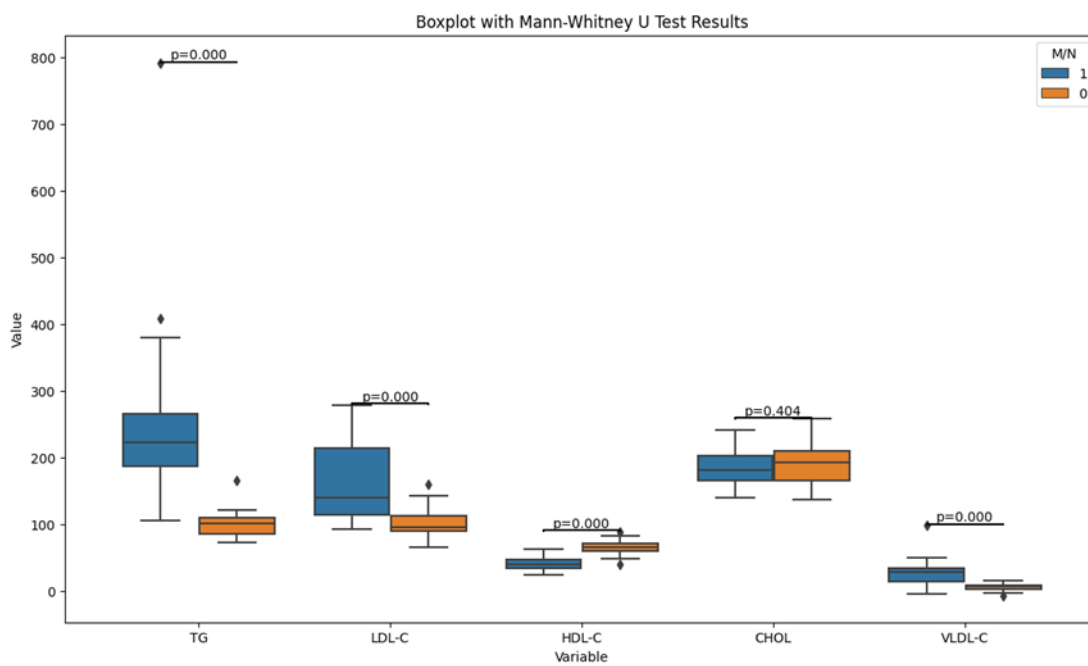

Supplement: S1 Appendix — (PDF) [file pone.0316522.s001.pdf]
