# Supplementary material for: Application of attenuated total reflection–Fourier transform infrared spectroscopy in semi-quantification of blood lipids and characterization of the metabolic syndrome
Source: PLoS One. 2025 Jan 30;20(1):e0316522. doi: 10.1371/journal.pone.0316522 (PMC11781649; doi:10.1371/journal.pone.0316522)

Appendix 2. Grouped spectrum with specified lipid value ranges

1. TG Spectrum

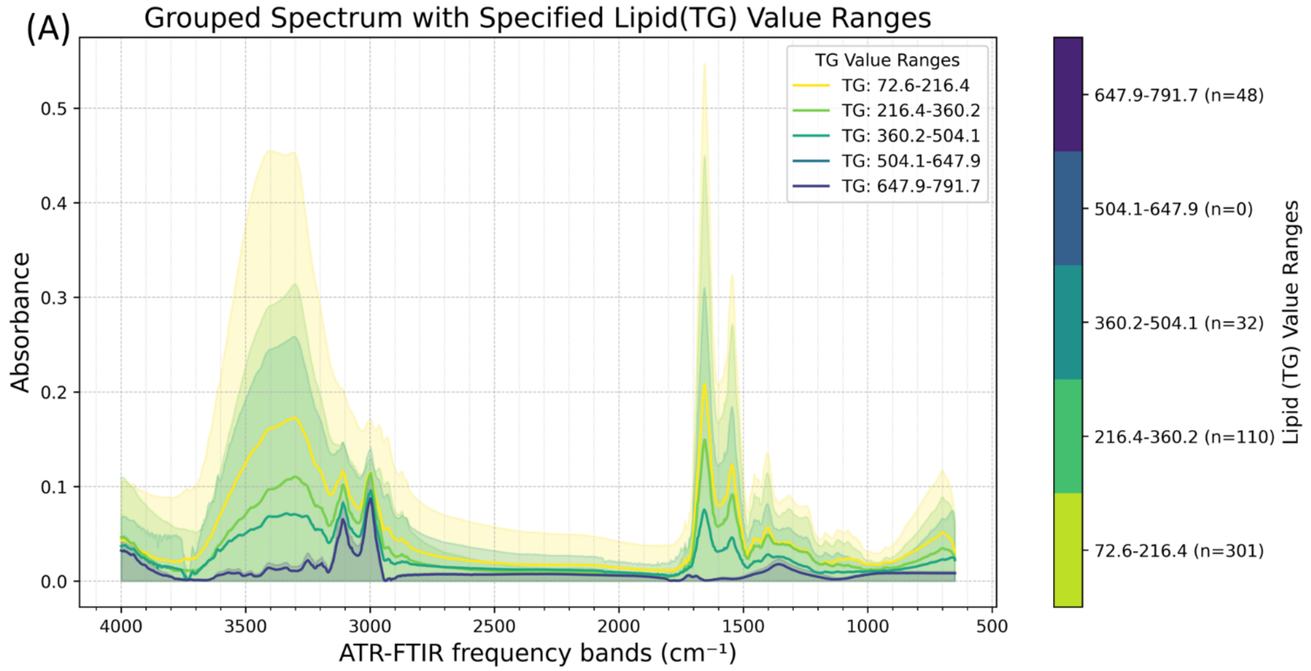

2. CHOL Spectrum

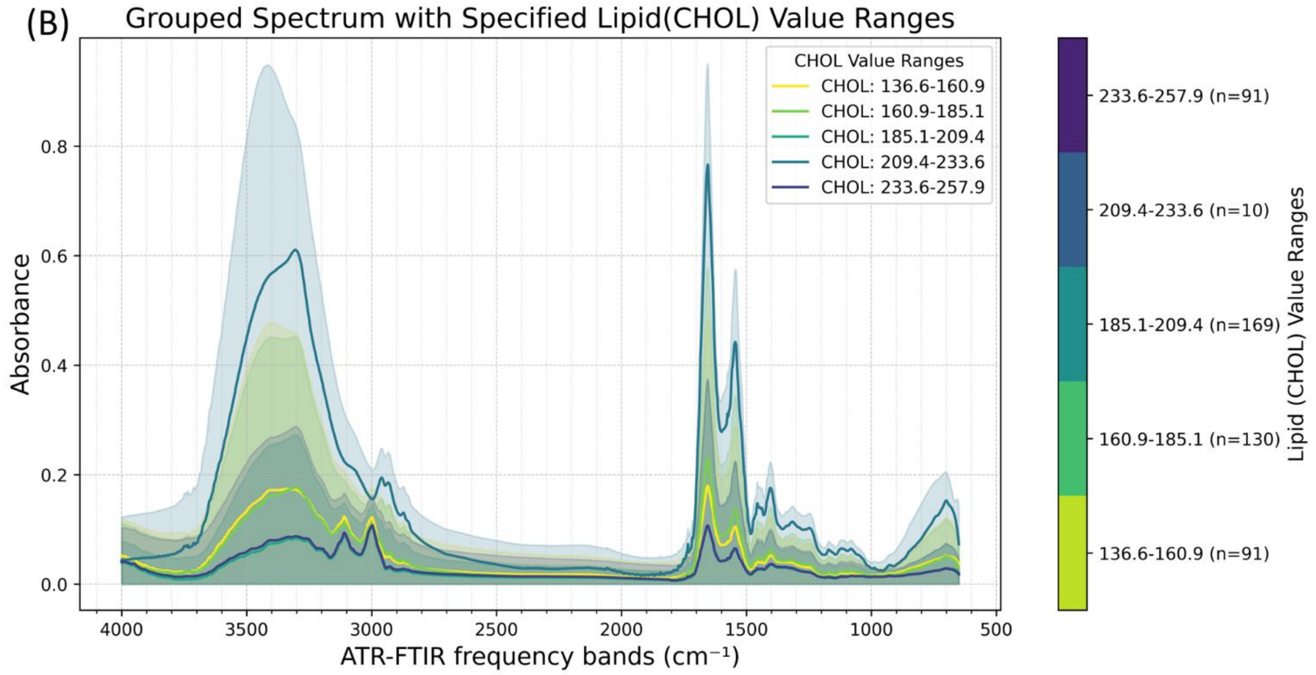

3. HDL-C Spectrum

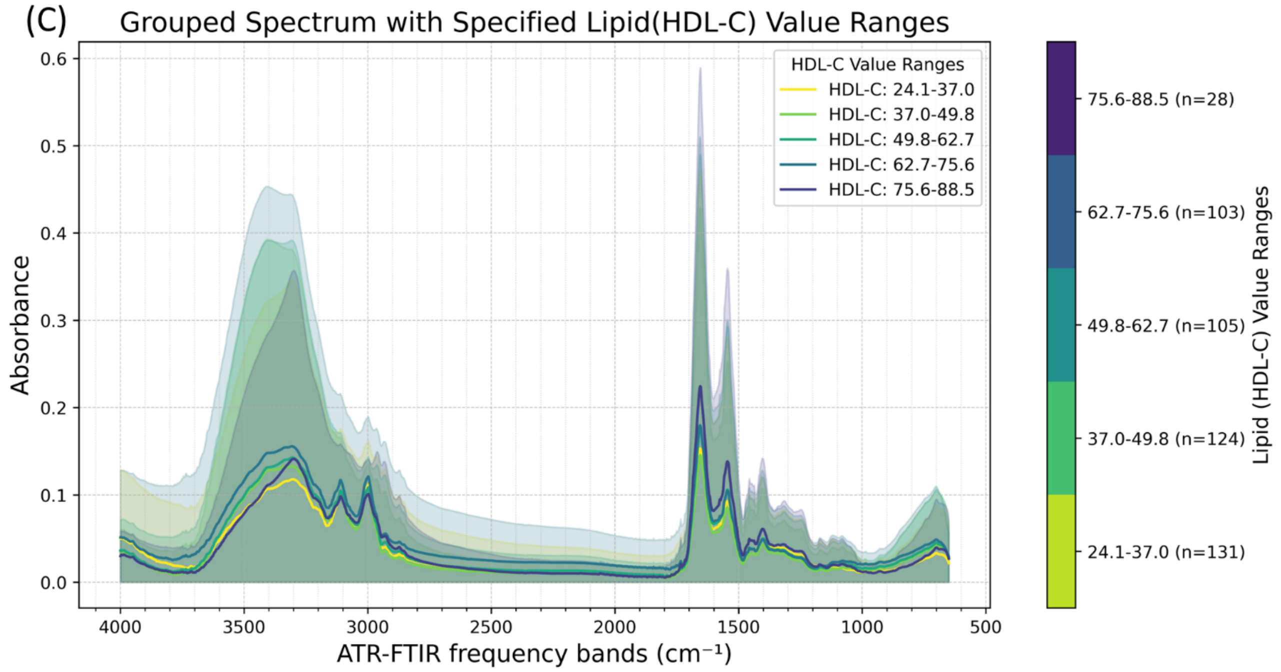

4. LDL-C Spectrum

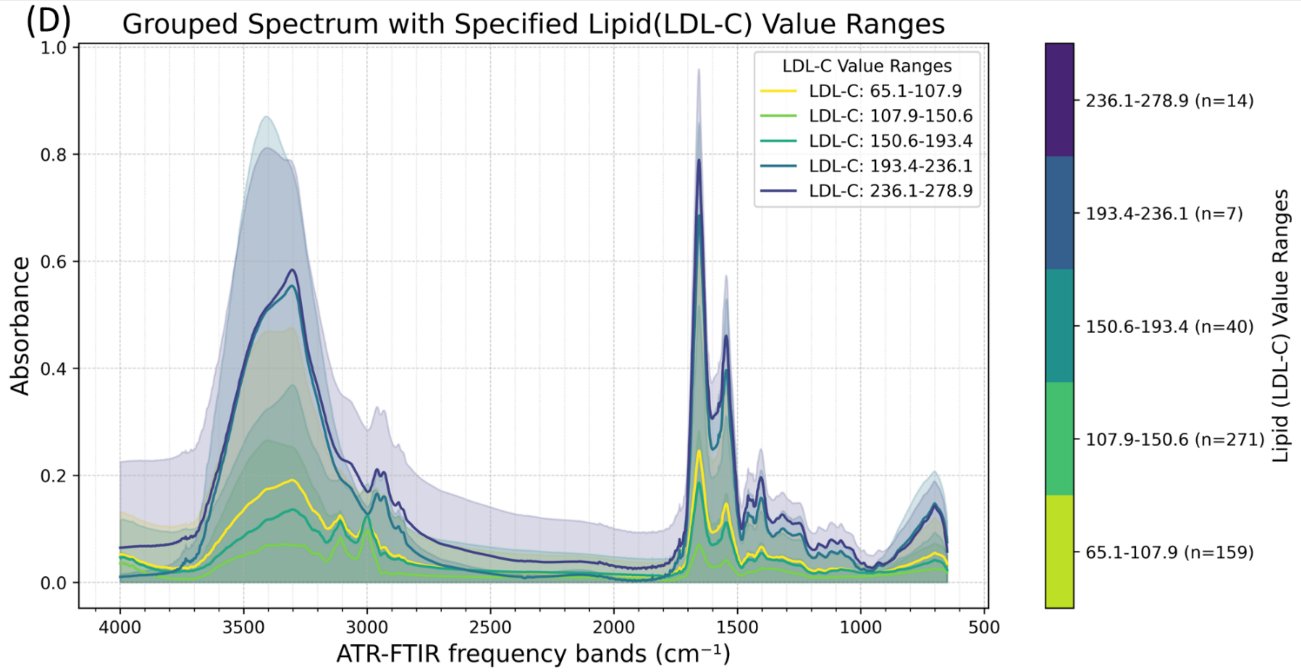

5.VLDL-C Spectrum

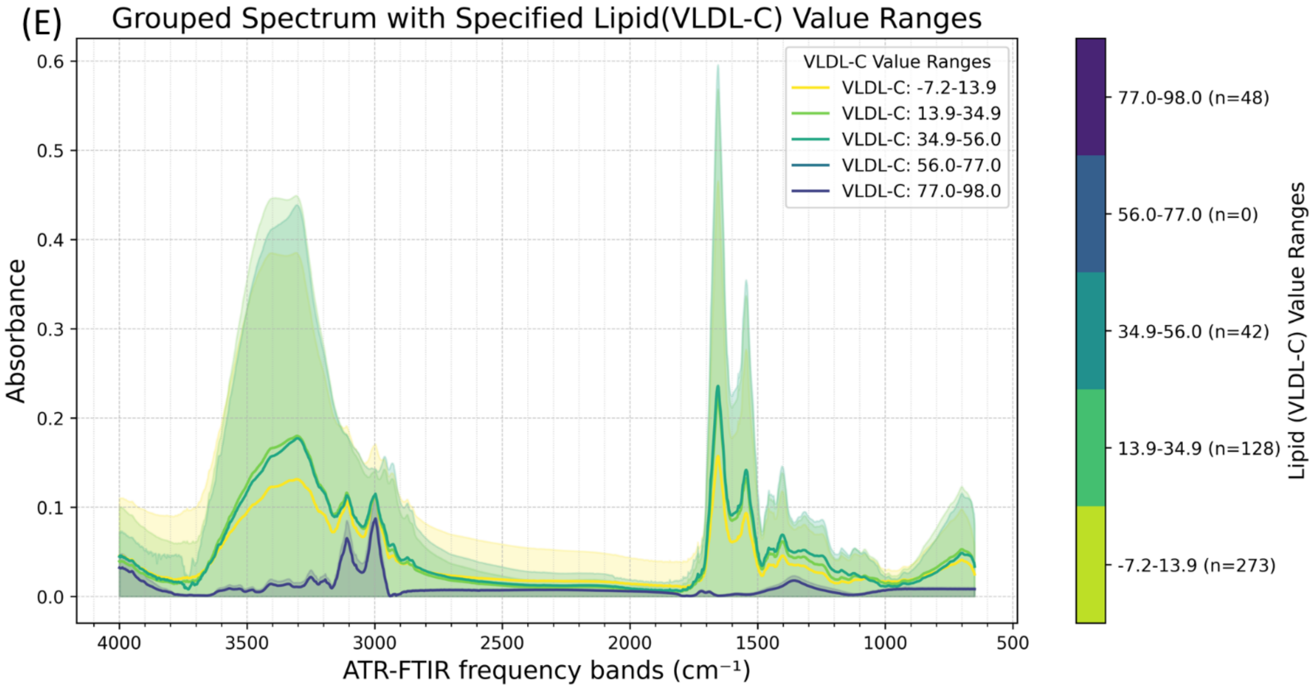

Supplement: S2 Appendix — (PDF) [file pone.0316522.s002.pdf]
