# Supplementary material for: Application of attenuated total reflection–Fourier transform infrared spectroscopy in semi-quantification of blood lipids and characterization of the metabolic syndrome
Source: PLoS One. 2025 Jan 30;20(1):e0316522. doi: 10.1371/journal.pone.0316522 (PMC11781649; doi:10.1371/journal.pone.0316522)

Appendix 3. Training deviance of the GBT model on different targets

We observed that during the cross-validation training and testing phases, the deviance typically decreased steadily across most folds. However, a few folds exhibited irregular patterns, likely due to the presence of outliers in the data distribution

Plot GBT models' training deviance of TG

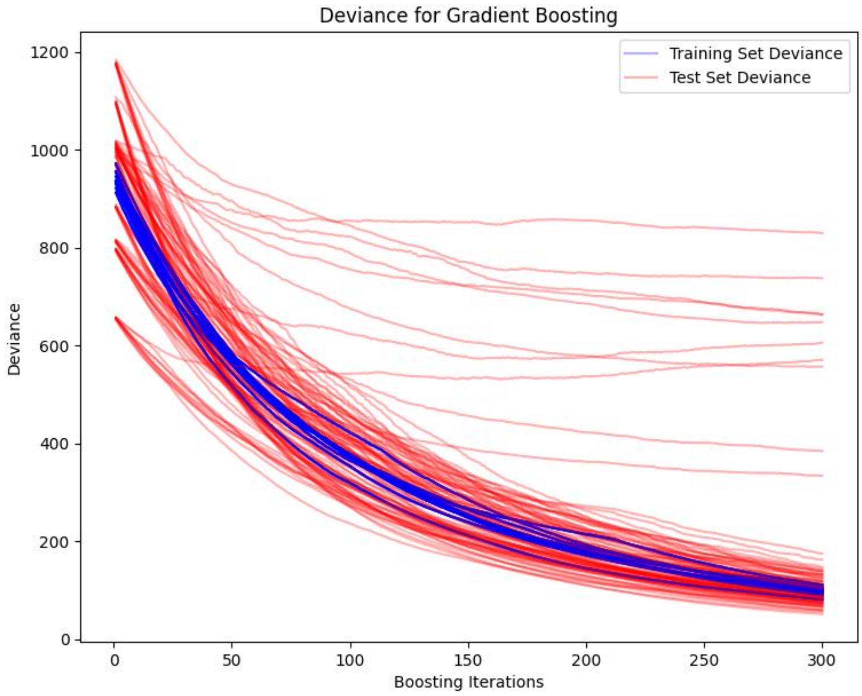

Plot GBT models' training deviance of LDL

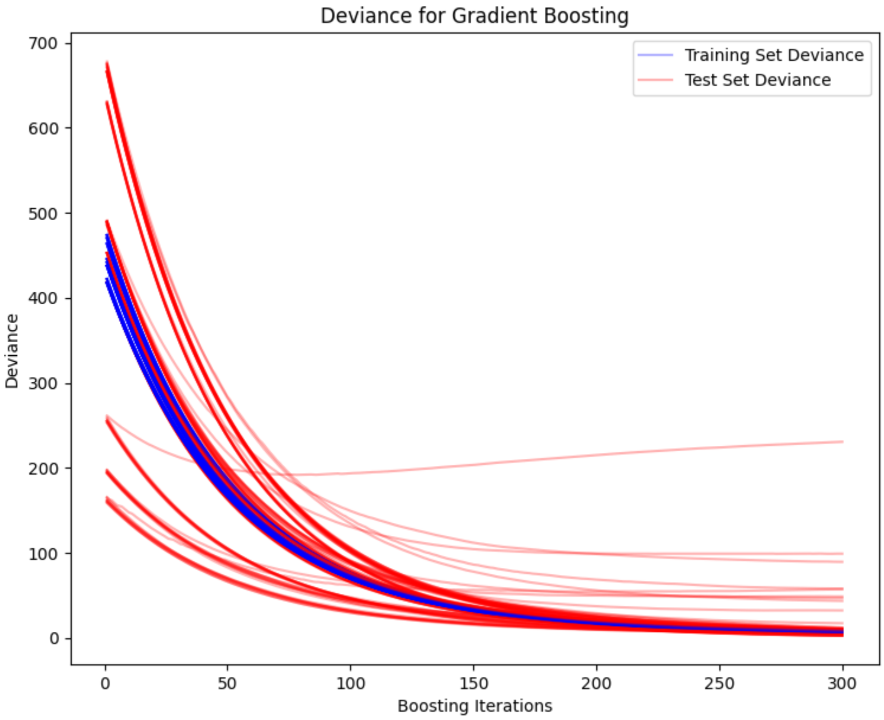

Supplement: S3 Appendix — (PDF) [file pone.0316522.s003.pdf]
