# Supplementary material for: Application of attenuated total reflection–Fourier transform infrared spectroscopy in semi-quantification of blood lipids and characterization of the metabolic syndrome
Source: PLoS One. 2025 Jan 30;20(1):e0316522. doi: 10.1371/journal.pone.0316522 (PMC11781649; doi:10.1371/journal.pone.0316522)

Appendix 4. The effect of the presence of MetS on the model's lipid values prediction

1. TG prediction

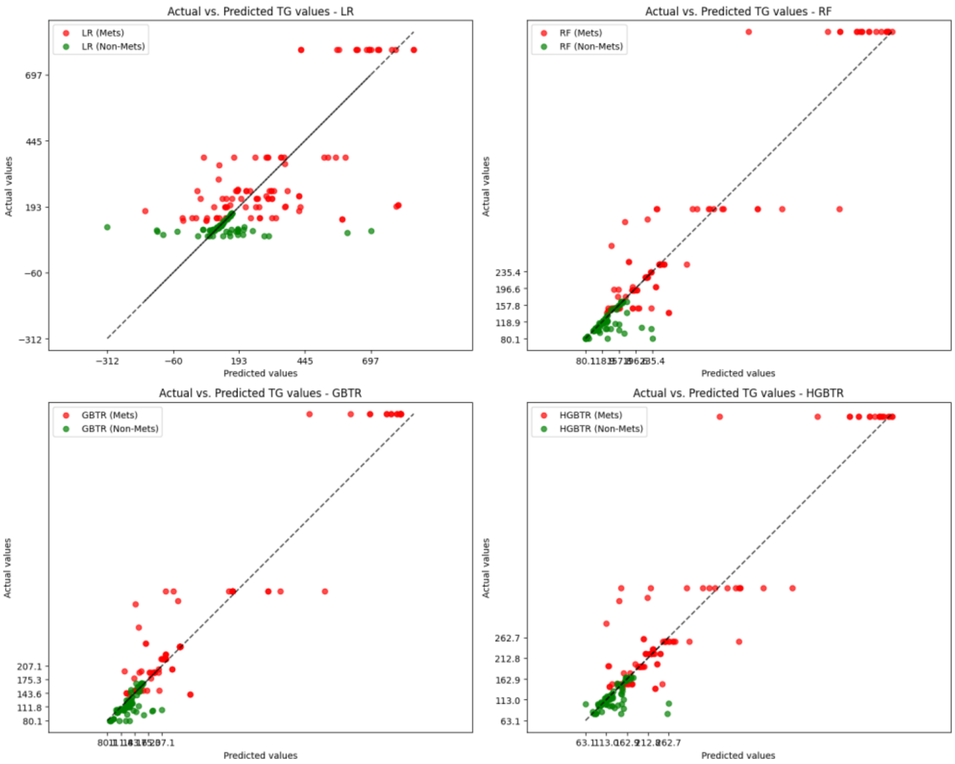

2. LDL-C prediction

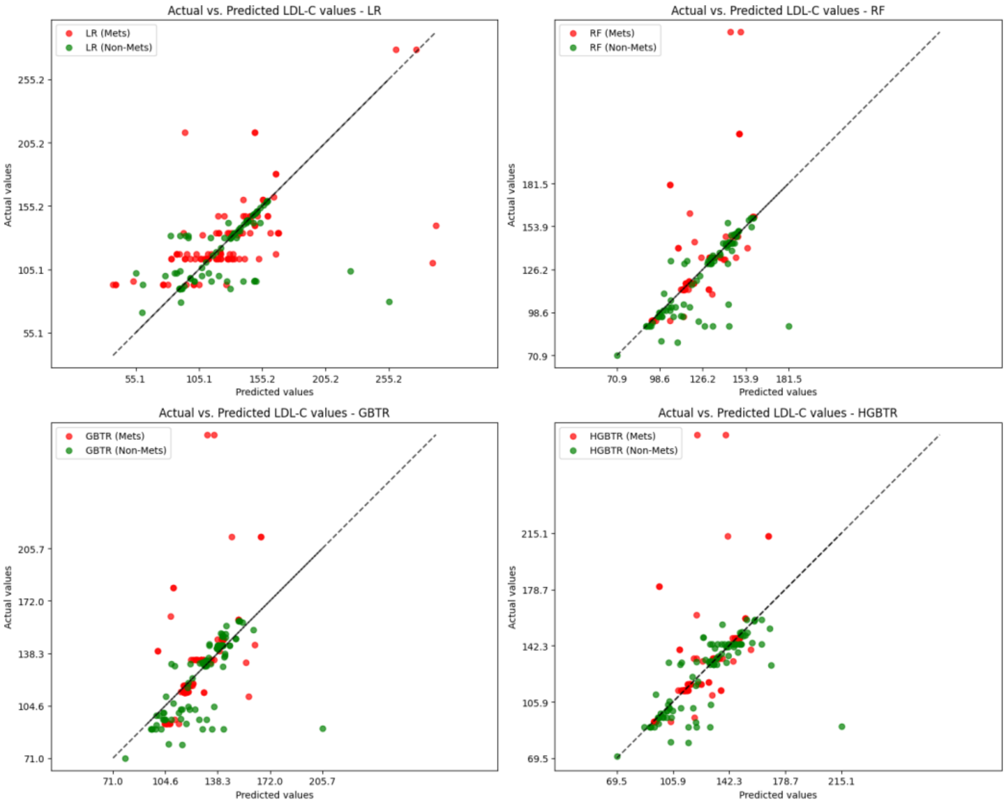

3. LDL-C (calculated by Friedewald formula) prediction

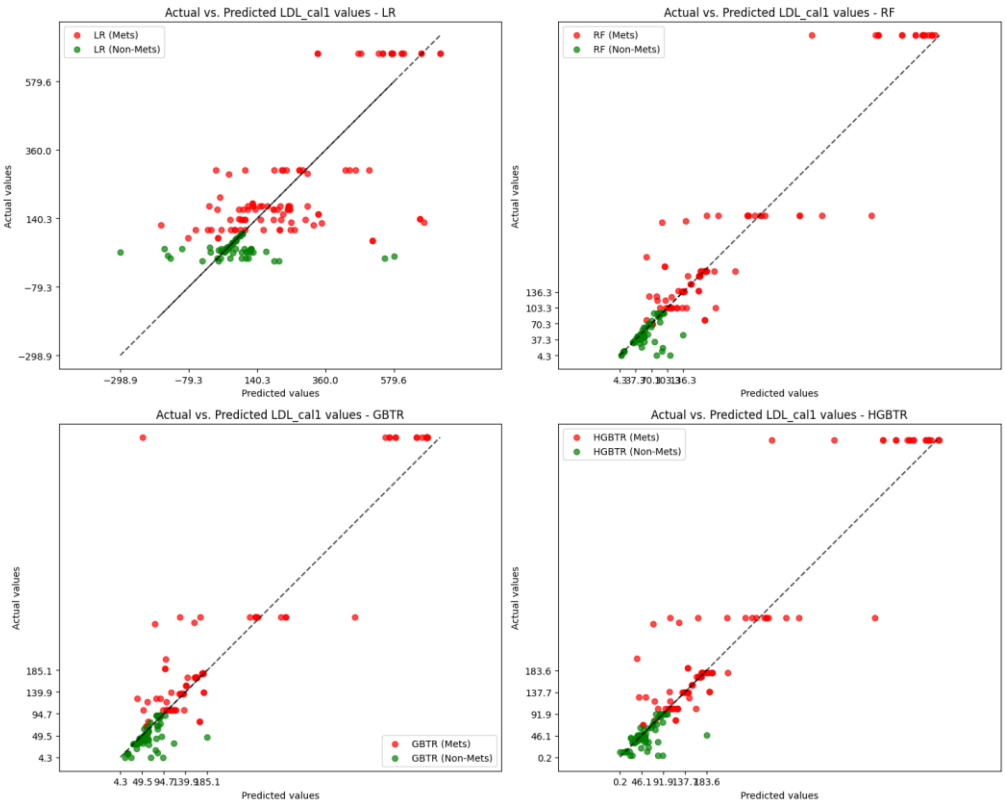

4. CHOL prediction

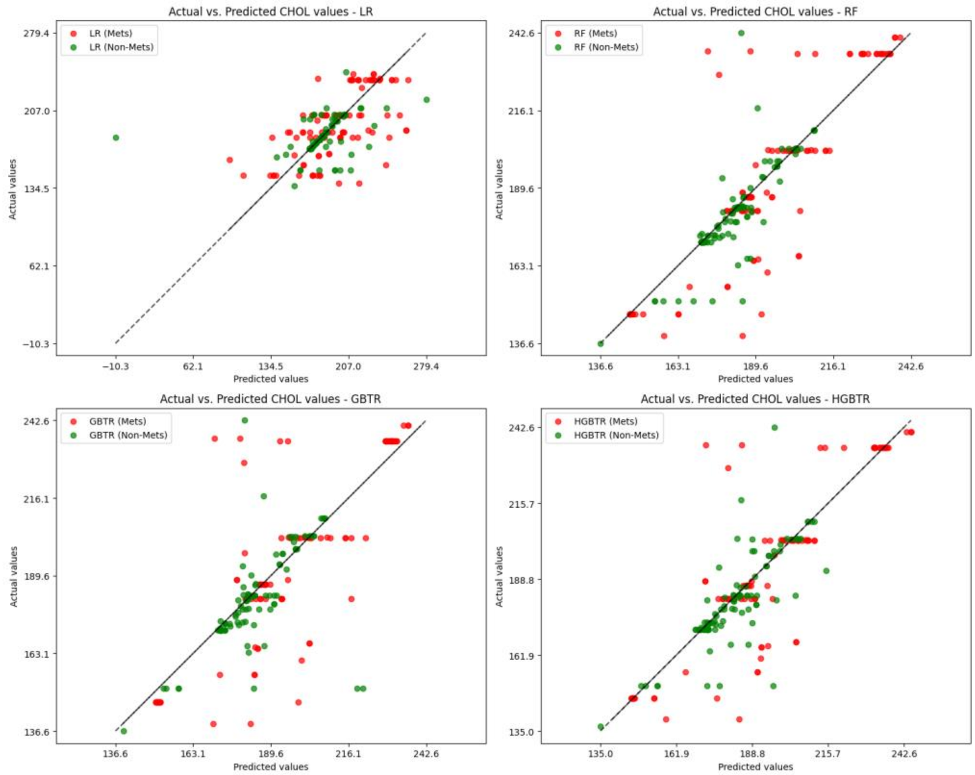

5. HDL-C prediction

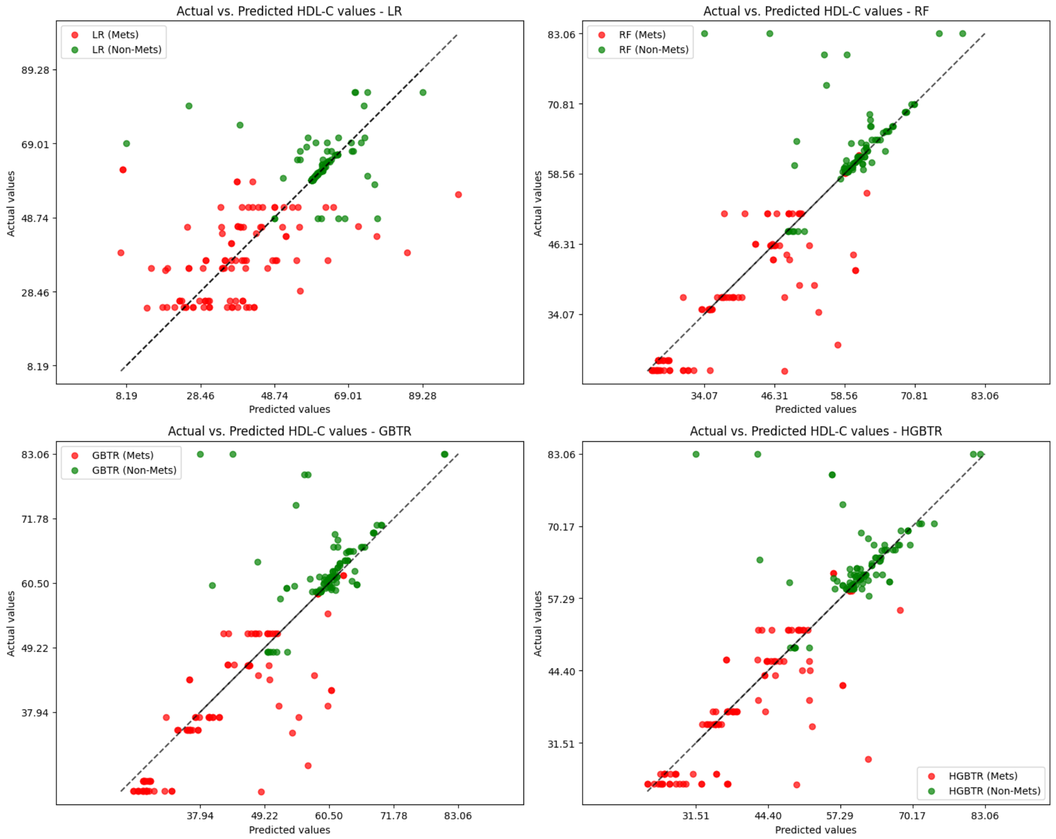

6. VLDL-C prediction

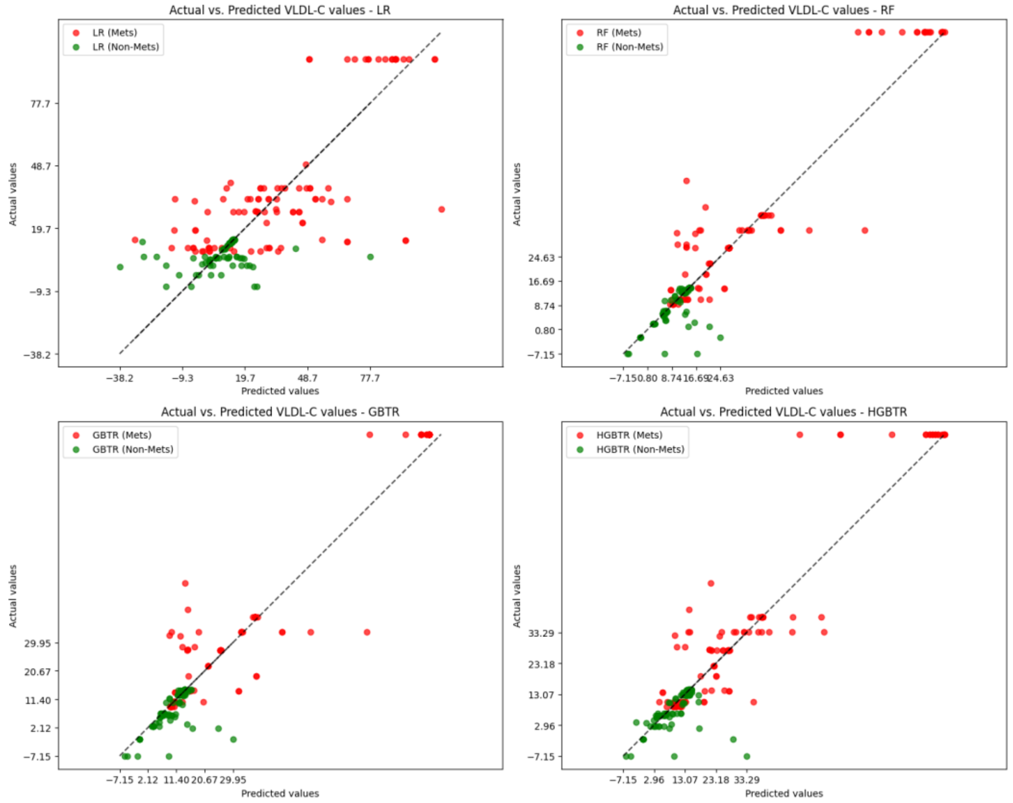

7.VLDL-C cross test

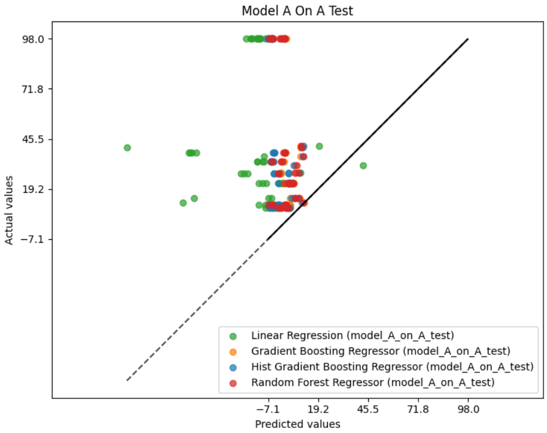

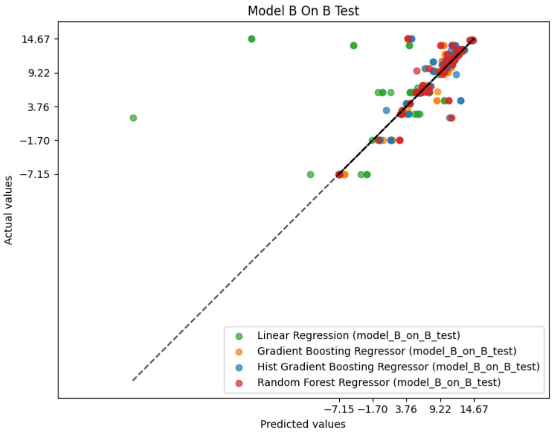

Supplement: S4 Appendix — (PDF) [file pone.0316522.s004.pdf]
