# Supplementary material for: Application of attenuated total reflection–Fourier transform infrared spectroscopy in semi-quantification of blood lipids and characterization of the metabolic syndrome
Source: PLoS One. 2025 Jan 30;20(1):e0316522. doi: 10.1371/journal.pone.0316522 (PMC11781649; doi:10.1371/journal.pone.0316522)
Supplement: Attachment — Submitted filename: Response to Reviewers.docx [file pone.0316522.s007.docx]

---------------------------------------------------------------

Specific Responses to the Reviewer Comments

---------------------------------------------------------------

Submission ID: PONE-D-24-11405

PONE-D-24-11405R1

Application of attenuated total reflection–Fourier transform Infrared spectroscopy in semi-quantification of blood lipids and characterization of metabolic syndrome

PLOS ONE

**Comments to the Author**

Reviewer #3: The study based on Application of attenuated total reflection–Fourier transform Infrared spectroscopy in semi-quantification of blood lipids and characterization of metabolic syndrome is highly significant. However I would suggest to authors please expand this study by using molecular methods.

**Response:**

Thank you for your comments. We agree that molecular methods are also very important for applying non-destructive ATR-FTIR spectrometry in serum analysis, particularly for lipids. The following studies demonstrate the use of ATR-FTIR spectrometry to analyze blood samples. Bel’skaya et al. published their work on using model solutions in applying FTIR spectroscopy for the quantitative analysis of blood serum.[32] Portaccio et al. published their study results on ATR-FTIR spectra of commercial lipid samples and demonstrated specific fingerprint regions in ATR-FTIR spectra for phospholipids (phosphatidylcholine, phosphatidylethanolamine, phosphatidylserine, and phosphatidylinositol) from 3500 to 2800 cm^−1^ and 1800 to 600 cm^−1^), sphingolipids (ceramide, sphingosine 1-phosphate, ceramide 1-phosphate, and sphingomyelin) from 3500 to 2800 cm^−1^ and 1800 to 600 cm^−1^), and cholesterol (3150–2800 cm^−1^) [33]. Their study proved the usefulness of FTIR spectra for analyzing complex lipids, and this application can ultimately contribute to lipid research and its implications for human health. (**Line 319-328**)

Based on the known advantage of FTIR spectra in quantitative analysis and sensitivity to differentiate various lipids, our study approach was directed toward linkage to clinical application. Consistent with the aforementioned literature, our results revealed the reliability of ATR-FTIR for semi-quantitative lipid analysis of human serum samples.

Unlike those publications, our study emphasizes the predictive capability of machine learning models. The core concept is to uncover the correlations between FTIR spectra, clinically derived lipid values, and diagnostic classifications.

Since this approach centers on data-driven correlations rather than direct molecular causation, it inherently lacks molecular specificity, which remains a limitation of ML-based predictions.

In future work, we may explore causation-focused models to investigate molecular associations further and validate these predictions. However, given the study’s current scope, our ML approach offers valuable insights into broad lipid level trends without requiring the extensive molecular characterization that lies beyond this study’s objectives. (**Line 380-384**)

Two new citations have been added.

32.	Bel’skaya LV, Sarf EA, Solomatin DV. Application of FTIR Spectroscopy for Quantitative Analysis of Blood Serum: A Preliminary Study. Diagnostics. 2021;11(12):2391. PubMed PMID: doi:10.3390/diagnostics11122391.

33.	Portaccio M, Faramarzi B, Lepore M. Probing Biochemical Differences in Lipid Components of Human Cells by Means of ATR-FTIR Spectroscopy. Biophysica. 2023;3(3):524-38. PubMed PMID: doi:10.3390/biophysica3030035.

Reviewer #4: • Provide additional context on ATR-FTIR: Add a brief explanation about how ATR-FTIR spectroscopy works, specifically for readers less familiar with the technique. This could improve the accessibility of the study’s technical approach.

**Response:**

Thank you for your comments. We have added a paragraph to **the Introduction and Methods** section to briefly explain how ATR-FTIR spectroscopy works. The context is as follows.

ATR-FTIR spectroscopy is a technique for analyzing the chemical composition of materials by detecting their characteristic absorption of mid-infrared light. In ATR-FTIR, molecules absorb mid-infrared light at specific wavenumbers (or frequencies), depending on their chemical bonds and structure. Each type of bond (e.g., C=O and C–H) has a unique absorption pattern, creating a molecular fingerprint. (**Line 77-81**)

This study applied a small sample volume (0.5 µL to a Ge ATR crystal, as shown in Figure 1(A). A modulated mid-infrared beam was directed into the Ge crystal, generating an evanescent wave at the crystal surface, allowing the light to interact with the sample. The sample absorbs light at characteristic wavenumbers, and the remaining light is detected using a liquid nitrogen-cooled mercury cadmium telluride (MCT) infrared detector. The resulting spectrum shows peaks corresponding to specific molecular bonds, providing a unique chemical profile of the sample. (**Line 122-127**)

Figure 1(A). The schematic depicts the attenuated total reflection for acquiring the FTIR spectra of a blood sample. The mid-infrared light is modulated using the Michaelson interferometer of an FTIR spectrometer. (**Line 142-144**)

• Add a visual comparison: Consider adding a figure that visually compares the ATR-FTIR spectrum with traditional spectra of individual lipid classes or a figure showing predicted vs. actual lipid levels for better visual interpretation.

**Response:**

Thank you for your comments. To illustrate the ability of spectral values to quantify traditionally categorized lipid classes (e.g., cholesterol and triglycerides), we categorized lipid values into five equally divided groups from maximum to minimum to examine potential spectral differences among varying lipid levels (see Figures below). The X-axis represents the input ATR-FTIR wavenumbers, the Y-axis represents the input ATR-FTIR spectral values, and the color group corresponds to the measurement group obtained from conventional biochemical assays. The average ATR-FTIR spectrum for each group is shown as a line, with the shaded regions representing plus one standard deviation. This visualization highlights the trends in spectral differences across lipid groups. However, although these trends suggest potential relationships, they do not allow for direct quantitative conclusions or inferences. Overlapping or minimal differences in FTIR spectral absorption across lipid ranges further suggest that nonlinear associations may exist that are not directly observable in raw spectral data. By applying machine learning models, we can efficiently capture and interpret these complex patterns, offering precise and meaningful lipid-level predictions beyond what is visually discernible. All figures are listed in **Appendix 2**. Grouped Spectrum with Specified Lipid Value Ranges (**Line 211-223**)

In response to your suggestion, we have also included Figure 2 to illustrate the comparison of predicted versus actual lipid levels. In this figure, the x-axis represents the lipid values predicted by the model based on the input ATR-FTIR spectral data, whereas the y-axis shows the actual measured lipid values. Additionally, we included a visualization of the distribution of error values to further elucidate the prediction accuracy and deviations of the model.

**1. TG Spectrum**

**2. CHOL Spectrum**

**3. HDL-C Spectrum**

**4. LDL-C Spectrum**

**5.VLDL-C Spectrum**
