# Supplementary material for: Application of attenuated total reflection–Fourier transform infrared spectroscopy in semi-quantification of blood lipids and characterization of the metabolic syndrome
Source: PLoS One. 2025 Jan 30;20(1):e0316522. doi: 10.1371/journal.pone.0316522 (PMC11781649; doi:10.1371/journal.pone.0316522)
Supplement: Attachment — Submitted filename: Specific Responses to Reviewers.docx [file pone.0316522.s006.docx]

---------------------------------------------------------------

Specific Responses to the Reviewer Comments

---------------------------------------------------------------

Submission ID: PONE-D-24-11405

**Editor’s Comments to Author**

Journal requirements:

When submitting your revision, we need you to address these additional requirements.

1. Please ensure that your manuscript meets PLOS ONE's style requirements, including those for file naming.

**Response:** Thank you for this comment. The manuscript has been checked and meets the PLOS ONE style requirements.

2. Please note that PLOS ONE has specific guidelines on code sharing for submissions in which author-generated code underpins the findings in the manuscript. In these cases, all author-generated code must be made available without restrictions upon publication of the work. Please review our guidelines at https://journals.plos.org/plosone/s/materials-and-software-sharing#loc-sharing-code and ensure that your code is shared in a way that follows best practice and facilitates reproducibility and reuse.

**Response:** Thank you for your comments. We accept the PLOS ONE guidelines regarding code-sharing and will provide the author-generated code (link at our Research Center website) upon publication of the work.

3. We note that the grant information you provided in the ‘Funding Information’ and ‘Financial Disclosure’ sections do not match. When you resubmit, please ensure that you provide the correct grant numbers for the awards you received for your study in the ‘Funding Information’ section.

**Response:** Thank you for your comments. We received funding to support this study and have updated the Funding Information with grand numbers on the submission page. One of our authors forgot to add a Financial Disclosure Statement before the primary submission. We are awaiting the journal’s approval for this correction.

4. In this instance it seems there may be acceptable restrictions in place that prevent the public sharing of your minimal data. However, in line with our goal of ensuring long-term data availability to all interested researchers, PLOS’ Data Policy states that authors cannot be the sole named individuals responsible for ensuring data access (<http://journals.plos.org/plosone/s/data-availability#loc-acceptable-data-sharing-methods>).

Data requests to a non-author institutional point of contact, such as a data access or ethics committee, helps guarantee long term stability and availability of data. Providing interested researchers with a durable point of contact ensures data will be accessible even if an author changes email addresses, institutions, or becomes unavailable to answer requests.

Before we proceed with your manuscript, please also provide non-author contact information (phone/email/hyperlink) for a data access committee, ethics committee, or other institutional body to which data requests may be sent. If no institutional body is available to respond to requests for your minimal data, please consider if there any institutional representatives who did not collaborate in the study, and are not listed as authors on the manuscript, who would be able to hold the data and respond to external requests for data access? If so, please provide their contact information (i.e., email address). Please also provide details on how you will ensure persistent or long-term data storage and availability.

**Response:** Thank you for your comments. Long-term data storage and availability for data requests are ensured by contacting the “Lipid Science and Aging Research Center (LSARC), Kaohsiung Medical University” (<https://lsarc.kmu.edu.tw/index.php/en-gb/overview/background>).TEL: +886-73121101 ext 2293.

**Reviewers' Comments to Author**

**Reviewer 1.**

1. After carefully reviewing the introduction section, I did not find a clear and explicit statement highlighting the novelty or newness of the study. The authors do not appear to directly state what makes their work novel compared to previous research in the field.

**Response:**

Thank you for your comments. We emphasize the novelty of our study, particularly highlighting the comprehensive insights into MetS provided by ATR-FTIR. Additionally, this is the first study to predict actual laboratory values with an acceptable MAPE by combining ATR-FTIR and different algorithms. (Line 291–294, 334–336, and 353–358)

2. The authors emphasize the advantages of their ATR-FTIR method, such as rapid assay times and the need for small blood samples (0.5 μL). These benefits make the method potentially better for frequent monitoring and clinical applications compared to established techniques. Unfortunately, the authors lack a comprehensive comparison of the accuracy, sensitivity, and specificity of their approach against established methods. A deeper analysis of the performance metrics and potential limitations of their technique, in relation to existing gold standards, would better support their case for the usefulness and innovation of their method.

**Response:**

Thank you for your comments and appreciation of the potential advantages of the ATR-FTIR method. Accordingly, statement was added in the study limitation as follows: “Fourth, verification using liquid chromatography–mass spectrometry (LC-MS) standards is lacking. The laboratory was approved by the Taiwan Accreditation Foundation for the fulfillment of ISO 15189 (Medical Laboratories Requirements for Quality and Competence), which supports the reliability of blood lipid concentrations. Future studies should focus on a comprehensive comparison of the accuracy, sensitivity, and specificity of the results from the AI-assisted FTIR spectrometry method with established methods using lipid standards.” (LINE 343–349). We made an amendment to Table 2 for the MAPE valves (LINE 226–231). In addition, we further investigated the effect of MetS on the lipid value predictions of the model (LINE 238–249) and added related figures in Appendix 3.

The mean absolute percentage error (MAPE) added to Table 2 and Metrics for model explanation, which is calculated by taking the average of the absolute differences between the predicted and actual values, divided by the actual values, and then expressing it as a percentage, can provide a clear and interpretable measure of prediction accuracy by expressing errors as a percentage of the actual values.

| MAPE(%) | TG | CHOL | LDL | HDL | VLDL |
| --- | --- | --- | --- | --- | --- |
| LM | 41.941 | 6.891 | 12.153 | 13.376 | 109.909 |
| RF | 12.252 | 3.393 | 7.329 | 6.902 | 38.043 |
| GBT | 14.057 | 3.744 | 10.045 | 8.087 | 42.149 |
| HGBT | 12.502 | 3.532 | 7.914 | 7.036 | 41.564 |

In our analysis, we observed that during the cross-validation training and testing phases, deviance typically decreased steadily across most folds. However, a few folds exhibited irregular patterns, likely owing to the presence of outliers in the data distribution (Appendix 2 for more information).

3. The conclusion should explicitly reiterate the potential impact of early detection and management of MetS on improving patient outcomes and quality of life, as highlighted in the introduction.

**Response:**

Thank you for your comments and the conclusion had been re-written as follows: This method provides a complete scout view of all MetS-related biochemical constituents, including all lipid species. Therefore, residual lipid-related risks can be revealed entirely. This comprehensive feature provides the potential for establishing an assessment system to stratify individuals with MetS, guide medical decisions, and monitor therapeutic effects. In conclusion, AI-assisted FTIR spectrometry blood sample analysis is worth future large-population studies to be applicable in clinical settings. (LINE 353–358)

4. What do you mean by the brackets in **table 2** as standard deviation (SD)?

**Response:**

Thank you for your comments. Subsequently, 10-fold cross-validation was used to calculate the mean and standard deviation (SD) of the above metrics across 10 models. A smaller standard deviation suggests that the performance of the model is relatively consistent across different folds. We hope that this clarifies this query.

5. However, there are a few minor points that could be improved in the reporting of the SHAP analysis: The authors could provide more details on how exactly the SHAP values were calculated (e.g., using the TreeExplainer for tree-based models like GBT) and what they represent (e.g., the contribution of each wavelength to the model's output relative to the average prediction). They could also discuss any potential limitations or caveats of using SHAP for model interpretation in this specific context.

**Response:**

Thank you for your comments. We appreciate the opportunity to provide more details and address any potential limitations of this study. The SHAP values represent the contribution of each feature (in our case, each wavelength) to the output of the model, relative to the average prediction (Fig 4). Essentially, SHAP values explain how a particular feature impacts the model's prediction, serving as a simplified, interpretable model to elucidate the workings of a more complex machine learning model. However, because SHAP uses a linear approach to explain nonlinear models, interpretability challenges can occasionally be introduced. Literature indicates that the range of 1645-1675 cm-1 primarily corresponds to non-cyclic carbon-carbon double bonds (C=C), which may be related to the structure of long-chain lipids in the blood. Notably, the combined contribution of the other 252 parameters was greater than that of the single wavelength at 1660 cm-1, indicating the advantage of using SHAP in FTIR spectrometry analysis. (LINE 327–336). Both Figure 4 and the figure legend have been revised accordingly (LINE 284–286).

6. From a purely statistical perspective, an R2 value of 0.419 indicates that only 41.9% of the variance in LDL-C levels can be explained by the ATR-FTIR spectral data using the HGBT model. This suggests that the model's predictions for LDL-C are not as accurate as those for other lipid parameters like TG (R2 = 0.854) or HDL-C (R2 = 0.758). In many scientific contexts, an R2 value below 0.5 would be considered poor model performance. I'm uncertain about the authors' argument, which claims that despite the low R² value, the mean absolute error (MAE) for LDL-C prediction was between 9 and 12 mg/dL.

**Response:**

Thank you for your comments. Thank you for your thoughtful feedback regarding the statistical interpretation of the R² value for LDL-C prediction using the ATR-FTIR spectral data with the HGBT model. We acknowledge that an R² value of 0.419 indicates that only 41.9% of the variance in LDL-C levels can be explained by our model, which may seem suboptimal compared to the R² values for other lipid parameters, such as TG (0.854) and HDL-C (0.758). However, we would like to provide additional context to clarify our argument regarding the model's performance.

The widely used Friedewald formula for calculating LDL-C is known to have inherent errors when compared with direct blood measurements, although it has been reported to be the most accurate way to calculate fasting LDL-C in Chinese people [newly added Reference #28]. For example, the MAPE and MAE for LDL-C calculated using the Friedewald formula were 77.5% and 92.3 mg/dL, respectively. In this study, our best model (Random Forest) achieves a significantly lower MAPE of 7.3% and an MAE of 9.7 mg/dL for LDL-C prediction. (LINE 226–231)

Our model achieved a considerably low prediction error (7.3 %) (Table 2). For instance, with an actual LDL level of 100 mg/dL, our model's prediction ranged from 93.7 to 107.3 mg/dL. Previous studies suggested a desirable bias of ± 6.8% and a desirable total allowable error (TEa) of ± 13.7% for LDL laboratory data and calculated errors by analyzing the European Federation of Clinical Chemistry and Laboratory Medicine database [35]. Therefore, the prediction error for LDL-C in this study did not affect the medication decisions or clinical judgment. (LINE 315–320)

We hope that this additional information clarifies our position and underscores the practical utility of our model in predicting LDL-C levels.

**Reviewer 2.**

(1) How were the samples stored?

**Response:** Thank you for this comment. The following statement has been added: “All ATR-FTIR spectral data were obtained at BL14A1 of the National Synchrotron Radiation Research Center (NSRRC) in Taiwan. To ensure sample stability, ATR-FTIR samples must be stored in a -80°C freezer prior to measurement and should be brought to room temperature naturally before measurement.“ (LINE 118–121)

(2) How did the authors ensure stability of samples?
(3) Was any heating of the sample applied prior to measurement? To prevent water interference?

**Response:** Thank you for this comment. The samples were placed on the ATR crystal surface to allow moisture to evaporate naturally before signal acquisition, ensuring that the moisture did not interfere with the spectra. If the moisture in the samples was not completely removed, interference appeared at 3500 cm-1, 2100 cm-1, and 1640 cm-1. A measurement time of approximately 5 min (~300 s) did not cause degradation of the components. (LINE 121–125). The samples were placed on the ATR crystal surface to allow moisture to evaporate naturally before signal acquisition, ensuring that the moisture did not interfere with the spectra. For further confirmation, we checked 3500 cm-1, 2100 cm-1, and 1640 cm-1 for possible water interference.

(4) Comment on transferability of the models to other matrices in the discussion

**Response:** Thank you for this comment. We have added new paragraphs to explain the potential applications of the models in the Discussion section. (Line 291–294 and LINE 353–358). We also add a new metric, the mean absolute percentage error (MAPE), which is calculated by taking the average of the absolute differences between predicted and actual values, divided by the actual values, and then expressed as a percentage, which can provide a clear and interpretable measure of prediction accuracy by expressing errors as a percentage of the actual values (LINE 163–169 and Table 2).

The MAPE values added in Table 2.

| MAPE(%) | TG | CHOL | LDL | HDL | VLDL |
| --- | --- | --- | --- | --- | --- |
| LM | 41.941 | 6.891 | 12.153 | 13.376 | 109.909 |
| RF | 12.252 | 3.393 | 7.329 | 6.902 | 38.043 |
| GBT | 14.057 | 3.744 | 10.045 | 8.087 | 42.149 |
| HGBT | 12.502 | 3.532 | 7.914 | 7.036 | 41.564 |

The following statement has been added. Fourth, verification using the standard of liquid chromatography–mass spectrometry (LC-MS) is lacking. The laboratory was approved by the Taiwan Accreditation Foundation for the fulfillment of ISO 15189 (Medical Laboratories Requirements for Quality and Competence), which supports the reliability of blood lipid concentrations. Future studies should focus on a comprehensive comparison of the accuracy, sensitivity, and specificity of the results from the AI-assisted FTIR spectrometry method with established methods using lipid standards. (LINE 343–349)
